# Hospital and Institutional News

**Published:** 1920-08-28

**Authors:** 


### HOSPITAL AND INSTITUTIONAL NEWS.

### DEATH OF SIR RILEY LORD.

At the age of eighty-two, after some months of indifferent health, Sir Riley Lord, ex-Mayor of Newcastle, and Chairman of the Finance Committee of the Royal Victoria Infirmary, Newcastle-on-Tyne, passed away on August 17 at his home in Northumberland. He was a man of great administrative ability and business capacity, who for over forty years was prominently associated with the work of the Prudential Assurance Company in the North of England. His experience in finance and his enthusiastic work on behalf of the Royal Victoria Infirmary have contributed no little to the growth and success of that institution. He was Chairman of the Building and Subscription Committees, and the building of the present premises (opened by the late King Edward) was made possible by the collection of a public fund of over £100,000 which he initiated, to which Lord Armstrong and the late Mr. John Hall each added £100,000. He took the keenest interest in everything pertaining to the development of the hospital, and it was for his signal services on its behalf that he was knighted in 1900. He was later made President of the institution, and was presented with an address by the Governors. He worked indefatigably in every direction to secure an adequate hospital for Newcastle, and extended his sympathy for the sick and suffering of the county to many other organisations. He was buried on August 20 at Elswick Cemetery, and a service was held in Newcastle Cathedral on the same day.

### THE NATIONAL RELIEF FUND AND THE HOSPITALS.

Since the early days of the war, when the accumulated funds of the National Relief Fund grew steadily larger without corresponding claims upon them, The Hospital has continuously urged the justice of the voluntary hospitals receiving a share of the receipts, seeing that their strained financial condition, even in those days, was undoubtedly due in part to circumstances arising from the war. The principle of making grants to hospitals was accepted by the National Relief Fund so far back as 1916, but the distribution of funds was confined to hospitals for soldiers. It is, therefore, with especial pleasure that we have received the announcement that the Committee of the Fund, after consultation with the Minister of Health, has decided to appropriate from the balance in hand (some £2,000,000 at June 30, 1919, the date of the last issued report) a sum of £700,000 towards meeting the outstanding liabilities of the voluntary hospitals of the United Kingdom incurred during the five years ended December 31, 1919, as a result of the war. The method of distribution is not yet settled, and a further announcement will be made, we are informed, towards the end of September. Meanwhile managers must curb their impatience, for no applications from individual hospitals will be dealt with. The National Relief Fund is shortly to be wound up, and particulars of the disposal of the unallocated balance will be awaited with interest. In any case, we congratulate the Committee of the National Relief Fund on their decision at last to recognise the hospitals as a proper channel for the distribution to the public of funds which were subscribed for the relief of distress due to the war.

### WHAT WILL BE THE METHOD OF DISTRIBUTION ?

Whether the National Relief Fund will act as its own almoner or not is yet undecided. Possibly the help of an outside body or bodies will be sought, and at first sight the British Red Cross Society commends itself to us in this connection as an important and competent agency. The Society has in hand a good deal of information as to the needs of the provincial hospitals and statistics for London, where, thanks to King Edward's Hospital Fund, balance sheets are prepared in a uniform manner and are easily procurable. The method of distribution is therefore to some extent plain. If the liabilities of a hospital were less at the end of 1919 than at the end of 1914 probably there would be no grounds for a grant. But then there is to be considered the question of deferred repairs and renewals, so important and pressing in most cases. The King's Fund in their emergency grant had to act quickly, which explains the fact that practically all the hospitals of London participated, although some of them were not in pressing need of help. There is more time for discrimination in the distribution of the Relief Fund.

### THE RED CROSS PEACE EFFORT.

It has been decided by the Joint Committee of the British Red Cross and St. John of Jerusalem to make a special effort during October and November for funds with which to carry out its work amongst the ex-soldiers and sailors who are still in need of treatment, and also to assist the Home Civil Hospitals and Curative Institutions—extension of Home Ambulance Service and Peace Library. It is hoped that the collections on one Sunday throughout the Kingdom can be donated to the joint societies, and an appeal urging this has been signed by the Archbishops of Canterbury and York, Cardinal Bourne, and the heads of all the Free Churches. The date selected is Sunday, November 14, and no better date could be chosen for such a purpose, following so closely as it does upon Armistice Day. Of course, if that day has been already allocated by any Church, then the next most convenient date is asked for. The amount from these Sunday collections will be shared with the Imperial War Relief Fund, which is dealing with the typhus scourge of Central Europe. A suggestion for combining hospital propaganda with the "Our Day" movement will be found elaborated in another column (p. 554).

### MRS. LLOYD GEORGE'S HOSPITAL WORK.

The King has been pleased to bestow the honour of Dame Grand Cross of the Order of the British Empire upon Mrs. Lloyd George. The honour, in the words of the official announcement, is given in recognition of the many services rendered by Mrs. Lloyd George during the war in connection with war charities and hospitals. In particular she helped to raise a sum of over £200,000. It is in Wales that Mrs. Lloyd George has naturally been most conspicuous, where she is identified with many institutions. The honour which she now receives has its own distinction, since it seems to have allowed every other claim to be considered first. It will give great pleasure to Welsh hospital workers, and indeed to all who welcome the recognition, so long in coming, of women's services to hospitals before and during the war.

### ADDENBROOKE'S AND UNDERGRADUATES.

The enlargement of Addenbrooke's Hospital, Cambridge, as a war memorial is having to wait for further support. The complete scheme will cost nearly £30,000, and towards this from every available source an assured sum of £17,000 is the utmost which can be relied on with certainty. This includes Red Cross grants, received or promised, and legacies earmarked for building. Mr. H. Head, the Secretary-Superintendent, and his Committee are thus placed in a difficulty, which is aggravated because there seems to be an impression that the hospital has already sufficient sums. Hospitals in university towns usually leave the undergraduates, except the handful of medical students, severely alone. Is this necessary? Social work of every kind is fashionable among the rank and file of both Universities. Why should not they be asked to take some interest in their local hospitals?

### THE NEW SECRETARY OF QUEEN'S HOSPITAL.

Mr. Hedley Lucas, Secretary and Superintendent of the Royal Albert Edward Infirmary, Wigan, has been appointed to the same office at the Queen's Hospital, Birmingham, which Mr. E. C. Smith is vacating on his appointment to the Bristol Royal Infirmary. Mr. Lucas's hospital experience extends to over thirteen years. After matriculating at the London University, he was for five years secretary to the Medical Superintendent at Monsall Fever Hospital; thence he went as clerk to Manchester Royal Infirmary; from Manchester as assistant secretary-superintendent at King Edward VII. Hospital, Cardiff; whilst the last five years have been divided between Stockport and Wigan, he having been successively secretary at each. It will be seen that Mr. Lucas's training and experience have been valuable, and he should prove to be a worthy successor at Birmingham to Mr. Smith. We congratulate both on their new appointments, which, we hope, will prove of pleasure and profit to themselves and the institutions they serve.

### THE SURGERY OF NERVES.

We would call attention to an extremely valuable "first report" just published by the Medical Research Council on the subject of injuries to the nervous system. The responsible committee has been presided over by Dr. Farquhar Buzzard and has included many world-famous men. Apart from the special interest which this subject holds for those interested in general, and particularly in industrial medicine, we see in this report a timely collecting of a number of war experiences, which in the changed circumstances of to-day might easily have been lost to the profession had not the Council foreseen the possibility. The great lesson contained by the report is that repair of nerve lesions is as much a matter of after-care as of actual operation. It is true that surgical technique has been improved in a high degree, giving more successful results than previous experience had led us to hope for, but nerve-grafting is not in any circumstances an easy operation, and the Committee advise it only when it is absolutely impossible to bring about direct approximation of the severed ends of the nerves. Their report itself contains an extraordinary amount of technical advice, and deals extensively with after-treatment and re-education, as well as operative procedures.

### INSTRUCTION IN VENEREAL TREATMENT.

A course has been arranged by the London (Royal Free Hospital) School of Medicine for Women, the Royal Free Hospital, the Elizabeth Garrett Anderson Hospital, and the London Lock Hospital, for the instruction of qualified medical women in the treatment of venereal diseases. The course will begin on Monday, September 13, at 12 noon, and will conclude on Saturday, September 25, 1920, extending therefore over two weeks. Qualified women wishing to attend are asked to send in their names to the warden and secretary of the Medical School not less than seven days before the course begins, the fee for which is £5 5s., payable on entry.

### THE STUDY OF HYGIENE.

The Department of Hygiene and Public Health at King's College, London, which offers complete courses of instruction for the various degrees and diplomas in public health, has recently been reorganised under the general supervision of Professor Simpson. Professor Sommerville lectures on hygiene, sanitary law and administration, sanitation and vital statistics, etc., and Mr. Rhys Charles on the Food and Drugs Acts. Bacteriology and parasitology is taught by Professor Hewlett and Dr. Taylor, and the chemical laboratory work is in charge of Mr. William Partridge. The laboratories are open daily for instruction and research, and arrangements are made to suit the convenience of those engaged in practice. Weekly demonstrations on sanitary appliances and visits to places of sanitary interest are arranged. A special course on industrial hygiene is given by Dr. Legge (October to February) and courses on school hygiene are given by Dr. Malcolm (October to June).

### MOSQUITOES.

Although there has been this year a very obvious profusion of newly-hatched individuals belonging to the eighteen different varieties of mosquito which Britain can claim, yet as far as we can ascertain there have been considerably fewer indigenous cases of malaria than last year, and nowadays these cases are very rare indeed. It is a point of interest when estimating the number of mosquitoes encountered in different localities that it is only the females which suck blood, the males feeding on the juices of plants. So much has been lately published on the technicalities of mosquito and mosquito larvæ destruction that the rural public should nowadays be placed in a position themselves to reduce the insects' numbers by no inconsiderable extent.

### GUARDIANS' HOSPITAL FOR PAYING PATIENTS.

The Barnet Board of Guardians is arranging to re-open the institution known as the Wellhouse Hospital for paying patients, as well as the sick poor. The building was planned in 1913 as a new infirmary for 192 beds, and the work was begun in January 1915, though the war made progress very slow. By March 1916, when it was half finished, the Guardians offered it to the Army Council on agreed conditions, and it was used as a war hospital till May 1919. The regulations are now being considered by the Ministry of Health, and include a clause for the admission of patients for whom "no suitable accommodation elsewhere can be readily obtained." This, which should cover any who can pay the full cost of maintenance and treatment, is stated to be based upon the opinion of the legal adviser to the Local Government Board in his evidence before the Royal Commission of 1906. Meantime the Guardians have appointed Dr. Stewart, the present medical officer, medical superintendent, and Dr. H. Chane Cox, formerly senior house surgeon at St. Bartholomew's Hospital, resident medical officer. The new matron has hitherto been matron of the Rotunda Hospital, Dublin. The Guardians express the strongest desire that the existing voluntary cottage hospitals in their district shall continue.

### DEVON AND THE MILITARY.

A vigorous and interesting report on voluntary aid work in Devonshire during and since the war has been issued, in which the County Director, Mr. J. C. S. Davis, goes fully into the work of each hospital, and gives a lively picture of the immense amount of voluntary work performed. Not the least interesting part of the report is a lengthy criticism of the military authorities, who are accused of having hampered the work in many ways and of having discriminated for no apparent reason against the organisation of this county and to have made things extremely difficult for the county director. The report remarks that a repetition of these experiences should be made impossible on the next occasion when the Devon Red Cross Branch is asked to provide V.A. hospitals.

### A WELSH AMBULANCE SERVICE.

Thanks to the Priory for Wales of the Order of St. John, good use is to be made of the motor-ambulance transport service called into being during the war. Medical and surgical cases have frequently to travel twenty miles or more to the nearest hospital, and it is proposed that the war service shall be maintained for such patients. Ultimately, it is planned to establish 175 stations throughout South and West Wales, connected by telephone with the police stations. A hopeful start has already been made in the coalfield, and of the eight cars allotted to Carmarthenshire two are at present in use at Llanelly and Ammanford. A systematic scheme of financial support from all trades and sections of the community has been devised, and the colliery owners have promised a sum of one shilling per employee per annum. The miners are asked to pay a penny a week per man, in return for which the ambulance cars will be at the disposal of their wives and children as well as of themselves. Many of the miners' lodges in South Wales have voted in favour of financial support.

### HELP FOR THE BLIND.

The second report of the Advisory Committee on the welfare of the blind has been issued by the Ministry of Health, and it contains much useful information respecting work for the blind in this country, which apparently is now largely in the hands of, and controlled by, the National Institute for the Blind and the Ministry of Health through its Advisory Committee. Registration of the blind, which should be completed next year by the help of the census, is proceeding satisfactorily. The number on the register on April 1, 1920, totalled 30,785, as compared with 25,840 early in the previous year. This does not mean that blindness has largely increased, but that the societies and other bodies who provide the information are coming forward with additional names hitherto unregistered. Reference is made in the report to proposals for grants-in-aid to approved agencies for the blind, and the Committee note with satisfaction that their recommendations have been accepted by the Government in this respect. The grants operated as from July 1, 1919, and are, as we have already announced, made only to charities which have been accepted for registration by the Ministry of Health and conform to its requirements as to the form of published accounts. We understand that the financial year has been fixed to end on March 31. The only clue to this rather awkward arrangement is apparently that the period coincides with the financial year of the national exchequer, although this, in itself, can hardly be put forward as a reason.

### THE DEMANDS OF THE BLIND.

Another combined effort is to be made by Boards of Guardians to induce the Government to take over and maintain the many hundreds of blind people now forced to seek Poor-law relief. It is doubtful whether the proposals of the Government to extend the old-age pensions to the blind will meet the situation, as many of them are receiving much larger amounts from the Guardians in the way of relief. It is certain that many blind people now seeking assistance by begging will not be satisfied with what they consider the inadequate amount of the old-age pensions, which is now limited to ten shillings a week. They earn much more by their precarious mode of living, and some are able to pay as much as thirty shillings a week to a person to lead them about. To meet their demands would mean a great annual expenditure, which it is doubtful whether any Government would be prepared to sanction. Some solution must be found to remedy the very unsatisfactory treatment of the blind.

### THE MINERS AND MERTHYR HOSPITAL.

The finances of the Merthyr General Hospital are again giving the governors some anxiety; there is a deficit of over £2,000 in current expenses. Moreover, the extension of the institution will be completed within the next six months, thus adding forty-five more beds (and more expenditure) to the total. Alderman F. T. James has suggested to the governors that one way out would be for the workmen of the borough to wipe off the debt by a special contribution of a shilling each. A better scheme, however, was indicated by Mr. B. J. Williams, one of the miners' representatives, who stated that he and his colleagues were at the moment engaged on a campaign to persuade the whole of the organised labour in the area served to fall into line with the steelworkers and the miners in paying a levy of four shillings a year towards the maintenance of the hospital. If Mr. Williams and his fellow governors are successful in their effort, not only will the debt be more than wiped out, but the hospital will be provided with an addition to its permanent income.

### NEW PROPOSALS AT EDMONTON INFIRMARY.

Edmonton Poor-Law Infirmary is the latest institution to be re-named. The Guardians have decided to call it in future Edmonton Municipal Hospital, and it is hoped that under the new title the scope of the hospital will be extended. The maternity work has largely increased and arrangements have been re-modelled; and co-operation with the local health authorities for the admission of private cases on a repayment basis has proved successful and is, in the opinion of the medical superintendent, likely to increase. In future the Hospital Committee is to be amalgamated with the House and Infirmary Committee under the title of the Hospital and House Committee.

### TWENTY-FIVE YEARS IN THE PUBLIC HEALTH SERVICE.

Dr. J. Priestley, Medical Officer of Health for Lambeth, has just completed twenty-five years' service as Medical Officer of Health under the late Lambeth Vestry and the present Lambeth Borough Council. The changes in the public service of a quarter of a century have been tremendous, but Dr. Priestley has kept abreast of the times. Many innovations have been initiated by him which later received the approval of the Government, and are now recognised to be essential to the public-health service of every borough.

### A NEW HOSPITAL PERIODICAL.

The Great Northern Central Hospital, Holloway, has begun the issue of a "Journal," called *Progress*, which is apparently to appear monthly, price threepence. The first number, which is well supplied with advertisements, gives an illustrated account of the foundation of the hospital and its branches at Clacton and Finchley, and of the different organisations connected with it. A recent garden fête is described in detail, accompanied by a page of photographs, and in this respect the Journal is like a magazine of hospital news. We shall be curious to see if there is enough news of this kind to fill a monthly periodical, since the object of *Progress* appears to be to bring to the notice of local readers all the hospital's affairs, and thereby to enforce the need of the extensions, and to encourage the people of the district to maintain it. The Ladies' Association and the League of Roses have their own pages. This sort of material is usually confined to local newspapers and special appeals; but we hope that *Progress* will justify itself and lead more people to realise what a centre of activity a hospital is, and how many opportunities it gives not only for useful service, but for making friendships, and filling life. Just as hospital gazettes appeal mainly to the staffs of the institutions, the new Journal seeks to attract all those who work for the hospital outside its walls and to attract their families and friends to the hospital's organisations. The attempt was worth making, and we wish it every success.

### AN ABUSE OF THE UNIFORM.

A curious effort has been made to raise money on behalf of the General Hospital at Nottingham. The idea, which originated with the manager of the tramway company, was that on a recent Saturday the cars should be boarded by "ladies dressed as Red Cross nurses" and members of the Ambulance Corps, who handed passengers envelopes wherein to place their contributions. On these envelopes the number of the ticket purchased was written, and at a subsequent draw the ticket-holder whose number is drawn will be entitled to a free week at Blackpool! The scheme produced £121. We do not wish to damp ardour, or to take a small matter too seriously, but the plan is one of those border-line cases which, if not actually objectionable itself, is insidious in example. Nor can we approve of people being "dressed" as nurses and boarding tramcars. The Red Cross collectors during the war were distinctively and attractively dressed without assuming the uniform. It is just as unsuitable for the nurse's uniform to be used for street collecting as for the soldier's; and we think that the General Hospital at Nottingham would have done better to rule out altogether this part of a well-intentioned but ill-conceived proposal.

### THIS WEEK'S DRUG MARKET.

Business continues extremely dull and there is no change in the tone of the market. Among articles which have receded in price is castor oil, but definite price reductions are few, although before business of any importance took place concessions would in some cases have to be made. Synthetic products continue very quiet, and there is still a tendency for prices to sag. Potassium bromide is offered at comparatively low rates. The improvement in the demand for and the price of menthol has been maintained. Nux vomica is again dearer. There is an improvement in the demand for eucalyptus oil and prices have a higher tendency.

